# Correction: Elford et al. Identification and Evaluation of Tools Utilised for Measuring Food Provision in Childcare Centres and Primary Schools: A Systematic Review. *Int. J. Environ. Res. Public Health* 2022, *19*, 4096

**DOI:** 10.3390/ijerph21050516

**Published:** 2024-04-23

**Authors:** Audrey Elford, Cherice Gwee, Maliney Veal, Rati Jani, Ros Sambell, Shabnam Kashef, Penelope Love

**Affiliations:** 1 School of Exercise and Nutrition Sciences, Institute for Physical Activity and Nutrition, Deakin University, Geelong, VIC 3216, Australia; elforda@deakin.edu.au; 2 Faculty of Health, University of Canberra, Bruce, ACT 2617, Australia; u3125198@uni.canberra.edu.au (C.G.); u3199404@uni.canberra.edu.au (M.V.); 3 School of Health Sciences and Social Work, Griffith University, Gold Coast, QLD 4222, Australia; r.jani@griffith.edu.au; 4 School of Medical and Health Sciences, Nutrition and Health Innovation Research Institute, Edith Cowan University, Perth, WA 6027, Australia; r.sambell@ecu.edu.au; 5 College of Nursing and Health Sciences, Flinders University, Adelaide, SA 5042, Australia; shabnam.kashef@flinders.edu.au

## Error in Figure

In the original publication [1], there was a mistake in Figure 1 as published. The original figure stated, “studies included in qualitative synthesis”. This is an inaccurate description of the extensive data extraction process that was undertaken. The figure should read: “82 studies included”. The corrected Figure 1 appears below.


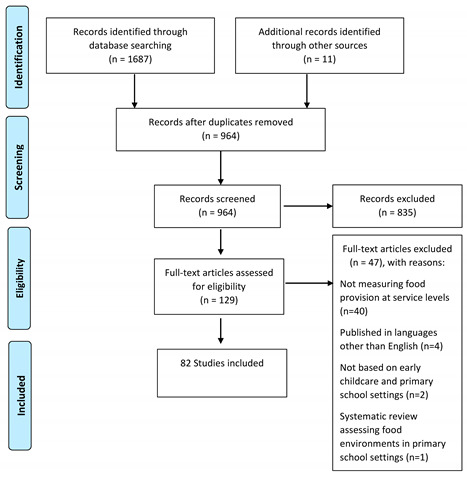


## Table Legend

In the original publication, there was a mistake in the legend for Table 1. The number of ECEC studies included was 47 not 45. The correct legend appears below.

**Table 1.** Summary of studies measuring food provision in ECEC settings (*n* = 47).

## Text Correction 1

There was an error in the original publication. Low–middle income countries (LMIC) should be High–middle income countries (HMIC).

A correction has been made to Introduction, Paragraphs 1 and 2:

Early childhood provides a unique window of opportunity to influence nutrition and dietary habits, as this is when food preferences and habits are formed, often tracking into adolescence and adulthood [1–3] and influencing health outcomes throughout the life course, in particular the risk of developing obesity [4]. Dietary patterns in High–middle income countries (HMIC) indicate that children’s dietary intakes do not meet nutrition guidelines, with an overall under-consumption of the core food groups, particularly vegetables and wholegrains, and over-consumption of discretionary foods, defined as processed foods high in fat, sugar and sodium [5–8]. According to the Global Burden of Disease Study across 195 countries, suboptimal dietary habits, (low intakes of wholegrains, fruit and vegetables and high intakes of sodium fat and sugar) account for more deaths than any other risk factor [9]. In addition, according to the World Health Organisation Global Strategy on Diet, Physical Activity and Health, the abovementioned dietary patterns, alongside sedentary behaviour, are the two main modifiable risks in the development of childhood overweight and obesity [10]. 

Instilling healthy dietary habits in childhood is therefore an important focus for public health interventions. While the home and family environments are regarded as primary settings to influence the dietary habits of children, early childhood education and care (ECEC) and primary school have become important environments for early intervention due to the significant time children spend in these settings. Approximately 50% of 3–5 year old children in HMIC countries are enrolled in ECEC [11,12] and almost 90% of children aged 6–11 years worldwide are enrolled in primary school [13,14]. Children enrolled in ECEC programmes in HMIC countries can spend up to five 8-h days each week in these settings [13,15,16], with average attendance rates being around 30 h per week in some countries [17,18]. Similarly, children enrolled in primary school, inclusive of Kindergarten up to Grade 6 [19], can spend up to five 7-h days per week in this setting [20,21]. Children attending ECEC and primary school therefore have a high level of exposure to external food environments for prolonged periods of time [15,22–24].

## Text Correction 2

There was an error in the original publication. 963 studies remaining after removal of duplicates should be 964 studies.

A correction has been made to Results, Paragraphs 1:

Database searching identified 1687 studies, with 964 studies remaining after removal of duplicates.

## Text Correction 3

There was an error in the original publication. Number of studies in ECEC settings should be 47 not 45.

A Correction Has Been Made to Results, 3.2. Description and Quality Appraisal of Studies, Paragraph 3:

Tables 1 and 2 provide detailed information regarding categorisation of identified measurement methods for the ECEC setting (*n* = 47) and primary school setting (*n* = 35).

The authors state that the scientific conclusions are unaffected. This correction was approved by the Academic Editor. The original publication has also been updated.
