# Supplementary material for: Identification and Evaluation of Tools Utilised for Measuring Food Provision in Childcare Centres and Primary Schools: A Systematic Review
Source: Int J Environ Res Public Health. 2022 Mar 30;19(7):4096. doi: 10.3390/ijerph19074096 (PMC8998327; doi:10.3390/ijerph19074096)
Supplement: Supplementary file 1 [file ijerph-19-04096-s001.zip › ijerph-1635144-supplementary.pdf]

Supplementary File 1: Quality assessment summary table

The quality rating is calculated as follows: Negative (-) indicates six or more questions are answered "no", Neutral (∅) if validity criteria questions (2, 3, 5 and 7) do not indicate the study is exceptionally strong and Positive (+) if most answers are yes including criteria 2, 3, 6, 7 and at least one other additional "yes" [36].

	First author, year	Quality rating	Validity Questions									
			Q1[a]	Q2[b]	Q3[c]	Q4[d]	Q5[e]	Q6[f]	Q7[g]	Q8[h]	Q9[i]	Q10[j]
1.	Agbozo, 2018	Positive	Y	Y	Y	N/A	UC	Y	Y	Y	Y	Y
2.	Aghdam, 2017	Positive	Y	Y	Y	N/A	N	Y	Y	Y	Y	Y
3.	Alves, 2015	Positive	Y	Y	N/A	N/A	N	Y	Y	Y	Y	UC
4.	Andreyeva, 2018a	Neutral	Y	Y	N	N/A	N	Y	N	Y	Y	Y
5.	Andreyeva, 2018b	Neutral	Y	Y	N	Y	N	Y	Y	Y	Y	Y
6.	Ball, 2007	Neutral	Y	UC	UC	N	N	Y	Y	Y	Y	Y
7.	Beets, 2015	Positive	Y	Y	N/A	N/A	N	Y	Y	Y	Y	Y
8.	Beets, 2017	Positive	Y	Y	Y	N/A	N	Y	Y	Y	Y	UC
9.	Bell, 2015a	Positive	Y	Y	Y	Y	N	Y	Y	Y	Y	Y
10.	Bell, 2015b	Positive	Y	Y	N/A	Y	N	Y	Y	Y	Y	N
11.	Benjamin-Neelon, 2010	Positive	Y	UC	N/A	N/A	Y	Y	Y	Y	Y	Y
12.	Benjamin-Neelon, 2013	Positive	Y	UC	N/A	N/A	UC	Y	Y	Y	Y	Y
13.	Benjamin-Neelon, 2015	Positive	Y	Y	Y	N	UC	Y	UC	Y	Y	Y
14.	Breck, 2016	Neutral	Y	N	N/A	N/A	N	Y	Y	Y	Y	Y
15.	Chriqui 2020	Positive	Y	Y	Y	N/A	N	Y	Y	Y	Y	Y
16.	Copeland, 2013	Neutral	Y	Y	N	Y	N	Y	Y	Y	Y	Y
17.	Dave, 2018	Neutral	Y	N	N/A	N/A	N	Y	Y	Y	Y	Y
18.	Davies, 2008	Positive	Y	UC	N/A	N/A	N	Y	Y	Y	Y	Y
19.	DeKeyzer, 2012	Neutral	Y	N	N/A	N/A	N	Y	N	Y	Y	N
20.	Dixon, 2016	Neutral	Y	Y	N/A	N/A	N	Y	N	Y	Y	Y
21.	Doak, 2012	Neutral	Y	UC	UC	N/A	N	Y	Y	Y	Y	Y
22.	Erinosho, 2011	Positive	Y	UC	N/A	N	N	Y	Y	Y	Y	Y
23.	Erinosho, 2013	Positive	Y	Y	N/A	N/A	N	Y	Y	Y	Y	Y
24.	Farris, 2014	Neutral	Y	N	N/A	N/A	N	Y	N	Y	Y	UC
25.	Finch, 2019	Positive	Y	Y	UC	Y	Y	Y	Y	Y	Y	Y
26.	Fleischhacker, 2006	Positive	Y	UC	N/A	N/A	N	Y	Y	Y	Y	Y
27.	Foster, 2015	Positive	Y	UC	N/A	N/A	N	Y	Y	Y	Y	UC
28.	Frampton, 2014	Neutral	Y	Y	N/A	N/A	N	Y	N	Y	Y	Y
29.	Gatenby LA, 2007	Neutral	Y	N	UC	N/A	N	Y	Y	Y	Y	Y
30.	Gerritsen, 2017	Neutral	Y	N	UC	N/A	N	Y	Y	Y	Y	Y
31.	Gougeon, 2011	Positive	Y	UC	N/A	N	N	Y	Y	Y	Y	Y
32.	Gregoric, 2015	Positive	Y	Y	N/A	N/A	N	Y	Y	Y	Y	Y
33.	Grady, 2019	Positive	Y	Y	N/A	N/A	N	Y	Y	Y	Y	Y
34.	Grady, 2020	Positive	Y	Y	Y	N	Y	Y	Y	Y	Y	Y
35.	Gurzo, 2020	Positive	Y	Y	N/A	N/A	N	Y	Y	Y	Y	Y
36.	Haroun, 2011a	Positive	Y	Y	N/A	N/A	N	Y	Y	Y	Y	Y
37.	Haroun, 2011b	Neutral	Y	UC	N/A	N/A	N	Y	N	Y	N	Y
38.	Hasnin, 2020	Positive	Y	N	N/A	N/A	N	Y	Y	Y	Y	Y
39.	Henderson, 2011	Positive	Y	Y	UC	Y	N	Y	Y	Y	Y	Y
40.	Himberg, 2019	Neutral	Y	N	N/A	N/A	N	Y	N	Y	Y	Y
41.	Huang, 2017	Neutral	Y	UC	N/A	N/A	N	Y	N	Y	Y	Y
42.	Ishdorj, 2016	Positive	Y	UC	N/A	N/A	N	Y	Y	Y	N	Y
43.	Jennings, 2011	Positive	Y	UC	N/A	Y	N	Y	Y	Y	N	Y
44.	Joyce, 2020	Positive	Y	Y	N/A	N/A	N	Y	Y	Y	Y	Y
45.	Kenney, 2015	Neutral	Y	N	N/A	N/A	N	Y	Y	Y	Y	UC

46.	Lassen, 2019	Positive	Y	Y	Y	N	UC	Y	UC	Y	Y	Y
47.	Lessard, 2013	Positive	Y	Y	Y	Y	Y	Y	UC	Y	Y	Y
48.	Longo-Silva, et al., 2013	Positive	Y	Y	Y	Y	UC	Y	Y	Y	Y	UC
49.	Martins, 2014	Positive	Y	Y	Y	Y	Y	Y	Y	Y	Y	UC
50.	Maalouf, 2013	Positive	Y	Y	N	UC	UC	Y	Y	Y	Y	Y
51.	Masis, 2017	Positive	Y	UC	UC	Y	UC	Y	Y	Y	Y	Y
52.	Morin, 2012	Positive	Y	Y	Y	N	UC	Y	Y	Y	N	UC
53.	Myers, 2019	Positive	Y	Y	Y	UC	UC	Y	N	Y	Y	N
54.	Myszkowska-Rcyiak, 2018a	Positive	Y	Y	Y	UC	UC	Y	UC	Y	Y	Y
55.	Myszkowska-Rcyiak, 2018b	Positive	Y	Y	Y	UC	Y	Y	N	Y	Y	Y
56.	Myszkowska-Rcyiak, 2019	Positive	Y	Y	Y	N/A	N	Y	N	Y	Y	Y
57.	Nathan, 2013	Positive	Y	Y	Y	Y	UC	Y	UC	Y	Y	Y
58.	Nathan, 2016	Positive	Y	Y	Y	Y	Y	Y	Y	Y	Y	Y
59.	Nicklas, 2013	Positive	Y	UC	Y	UC	Y	Y	Y	Y	Y	Y
60.	O'Halloran, 2018	Positive	Y	Y	Y	N	UC	Y	Y	Y	Y	Y
61.	Ohri-Vachaspati, 2012	Neutral	Y	Y	Y	N	UC	Y	Y	Y	UC	Y
62.	Parker, 2011	Positive	Y	Y	Y	N	UC	Y	Y	Y	Y	Y
63.	Patterson, 2013	Neutral	Y	Y	Y	UC	UC	Y	UC	Y	Y	Y
64.	Pearce, 2011	Neutral	Y	UC	UC	Y	UC	Y	Y	Y	N	Y
65.	Pearce, 2013	Neutral	Y	UC	Y	UC	UC	Y	Y	Y	N	N
66.	Perez-Ferrer, 2018	Positive	Y	Y	Y	Y	UC	Y	Y	Y	Y	Y
67.	Reilly, 2016	Positive	Y	Y	Y	N	Y	Y	Y	Y	Y	UC
68.	Reilly, 2018	Neutral	Y	N	Y	UC	UC	Y	Y	Y	Y	UC
69.	Retondario, 2016	Positive	Y	Y	Y	Y	UC	Y	Y	Y	Y	Y
70.	Romaine, 2007	Neutral	Y	Y	Y	N	UC	Y	UC	Y	Y	UC
71.	Sambell, 2019	Positive	Y	Y	Y	Y	UC	Y	Y	Y	Y	Y
72.	Schwartz, 2015	Positive	Y	Y	Y	Y	Y	UC	Y	Y	Y	Y
73.	Taylor, 2014	Positive	Y	UC	Y	UC	Y	Y	Y	Y	Y	Y
74.	Turner, 2016	Positive	Y	Y	Y	Y	UC	Y	Y	Y	Y	Y
75.	Turner-McGrievy, 2014	Positive	Y	UC	Y	UC	UC	Y	Y	Y	Y	Y
76.	Vieux, 2018	Positive	Y	Y	Y	UC	Y	Y	Y	Y	Y	N
77.	Vossenaar, 2011	Neutral	Y	UC	UC	UC	UC	UC	Y	Y	Y	Y
78.	Vossenaar, 2015	Positive	Y	Y	Y	UC	UC	UC	Y	Y	Y	Y
79.	Ward, 2017	Positive	Y	Y	Y	Y	UC	Y	Y	Y	Y	Y
80.	Weber, 2010	Neutral	Y	UC	UC	UC	Y	Y	Y	Y	Y	Y
81.	Woods, 2014	Positive	Y	Y	Y	UC	UC	Y	Y	Y	Y	Y
82.	Yoong, 2019	Positive	Y	Y	Y	UC	Y	Y	Y	Y	Y	Y

Abbreviations: Y = yes; N = no; UC = unclear; N/A = not applicable

[a]Q1=Question 1: Was the research question clearly stated?

[b]Q2=Question 2: Was the selection of study subjects/patients free from bias?

[c]Q3=Question 3: Were study groups comparable?

[d]Q4=Question 4: Was method of handling withdrawals described?

[e]Q5=Question 5: Was blinding used to prevent introduction of bias?

[f]Q6=Question 6: Were intervention/therapeutic regimens/exposure factor or procedure and any comparison(s) described in detail? Were intervening factors described?

[g]Q7=Question 7: Were outcomes clearly defined and the measurements valid and reliable? *[Note: for the purposes of this review, a 'Yes' was recorded if either validity or reliability was reported. The issue of limited validity and reliabilty is explored further in the manuscript.]*

[h]Q8=Question 8: Was the statistical analysis appropriate for the study design and type of outcome indicators?

[i]Q9=Question 9: Are conclusions supported by results with biases and limitations taken into consideration?

[j]Q10=Question 10: Is bias due to study's funding or sponsorship unlikely?

Reference: Academy of Nutrition and Dietetics. Quality criteria checklist: primary research in evidence analysis manual: steps in the academy evidence analysis process. Available online: http://andevidencelibrary.com/files/Docs/2012_Jan_EA_Manual.pdf
